# Can Caregivers Forecast Their Child’s Postoperative Disability After Elective Orthopedic Surgery?

**DOI:** 10.7759/cureus.48575

**Published:** 2023-11-09

**Authors:** Danika Baskar, Shayna Mehta, Halle Freiman, Nicole A Segovia, Brian B Vuong, Ann Richey, Joanna L Langner, Katherine G Hastings, Robin N Kamal, Steve Frick

**Affiliations:** 1 Orthopaedic Surgery, Stanford University School of Medicine, Palo Alto, USA; 2 Orthopaedic Surgery, Stanford University School of Medicine, Stanford, USA

**Keywords:** orthopedic surgery, pediatric, function, disability, pain, outcomes, postoperative, catastrophizing, caregiver, parent

## Abstract

Background

While there has been a growing emphasis on evaluating the patient’s perspective of health outcomes, caregiver expectations of post-orthopedic procedure disability and pain in a pediatric population are yet to be investigated. This study evaluates whether caregivers’ preoperative expectations of pain and function differ from their child’s early outcomes after surgical orthopedic intervention.

Methodology

Patients eight to 18 years old undergoing elective orthopedic surgery were enrolled. The caregivers of consented patients completed a survey at the child’s preoperative appointment to predict their postoperative pain and disability. The child was given the same survey during their postoperative visit four to six weeks after surgery to assess actual levels of functioning following the procedure. Scores were analyzed to study correlations between patient and caregiver responses (n = 48).

Results

Caregivers underestimated their child’s postoperative psychosocial functioning, as evidenced by the Psychosocial Health Summary Score, and overestimated pain, as demonstrated by the Numeric Pain Rating Scale. The Pediatric Quality of Life Inventory scores showed caregivers had differing expectations of the impact surgery had across various aspects of the physical, emotional, social, and school functioning domains. Higher parental pain catastrophizing was associated with underestimated predictions of their child’s psychosocial functioning after surgery. No significant difference was found in the patient’s physical functioning, as shown by the Physical Health Summary Score.

Conclusions

Surgical intervention is a major event that can provoke anxiety for parents and caregivers. Understanding differences in caregiver perspectives and early postoperative patient outcomes provides physicians valuable insights. Explaining to caregivers that patient psychosocial factors and functional outcomes after surgery are commonly better than expected can alleviate anxiety and prevent catastrophizing. This knowledge can help guide caregiver expectations and plans for their child’s postoperative pain control and functional recovery.

## Introduction

The evaluation of psychological and social health determinants, and how they impact patient outcomes, has been an area of growing interest. Research continues to demonstrate the importance of considering these factors in care planning and further emphasizes the need to adopt a biopsychosocial approach to providing support [1]. The biopsychosocial health model was first introduced in 1977 by George Engel who described the impact psychosocial factors, including emotional states, patient beliefs, behaviors, and social impact, can have on affecting human biology and disease [2]. This multidisciplinary approach allows for various aspects of a patient’s health and treatment to be addressed and has gained widespread acceptance across several healthcare disciplines in consideration for designing comprehensive interventions.

Chronic disease and musculoskeletal pain literature have shown that addressing psychosocial factors can aid in improving the overall burden of disease and disability as the experience and intensity of symptoms may be accounted for by one’s mental and social health [1-3]. Considering patients who undergo surgery, evidence from studies investigating the effect of psychosocial health determinants on a patient’s functional outcomes after orthopedic intervention similarly highlights the need to proactively address these concerns given their impact on postoperative function and recovery [4]. Other domains that have been found to significantly affect a patient’s functional outcomes include depression, anxiety, catastrophic thinking, and low self-efficacy [5,6]. While this has been characterized among adults undergoing operative treatment of the knee, spine, hip, and shoulder, it remains an area that is yet to be studied among pediatric patients [5-9].

The dynamic between a parent and their child is one that is unique among pediatric surgical disciplines and adds an additional influence in comparison to adults undergoing surgery. A child’s family environment has been shown to affect functional engagement, pain response, and their ability to adhere to medical recommendations [10]. Parental factors have also been found to impact their child’s experienced pain, symptoms, and treatment outcomes, and can be linked to certain behaviors including caregiver pain catastrophizing and protective responses [11]. This has partially been attributed to the child’s social learning ability, where they may experience a similar response after observing parental reactions to emotional situations involving fear, anxiety, and sensitivity [12]. Considering orthopedic surgical intervention can be a source of significant stress for a child and their family, an emotional response to this event is to be expected and can be appropriately addressed during the preoperative visits. This study aims to understand how caregivers’ preoperative expectations of pain and function differ from their child’s early outcomes after surgical orthopedic intervention. Identifying domains of dissonance can aid in the development of targeted interventions to proactively guide caregiver expectations of their child’s postoperative pain and disability.

This article was previously presented as a meeting abstract at the Western Orthopaedic Association (WOA) meeting in August 2021, the American Academy of Orthopaedic Surgeons (AAOS) meeting in March 2022, and the Pediatric Orthopaedic Society of North America (POSNA) meeting in May 2022.

## Materials and methods

In this prospective cohort study, a total of 48 patients between the ages of eight and 18 years undergoing elective orthopedic surgery were recruited from faculty division practices for enrollment in the study. Based on a power analysis, a sample size of 46 provided at least 80% power to detect a 20% difference in outcomes. Caregivers of consenting patients were asked to complete a survey at their child’s preoperative appointment to predict their postoperative pain and disability. The survey consisted of a battery of assessments including the Physical Health Summary Score (PHS), Psychosocial Health Summary Score (PSHS), Numeric Pain Rating Scale (NPRS), Pediatric Quality of Life Inventory (PedsQL), and the Pain Catastrophizing Scale (PCS). The PedsQL comprises three summary scores, namely, a total scale score, a physical health summary score, and a psychosocial health summary score, that measure physical, emotional, social, and school functioning. The child was given the same survey during their postoperative visit four to six weeks after surgery to assess actual levels of functioning following their procedure. Scores were analyzed, and responses were compared to study correlations between caregivers’ predictions of their child’s postoperative disability to the patient’s actual functioning after surgery. Statistical analysis was performed using two-sample t-tests and Mann-Whitney tests with a level of significance of 0.05 to draw correlations between caregiver predictions and the child’s postoperative outcomes. All study activities were approved by the Stanford University School of Medicine Institutional Review Board under protocol number 46548.

## Results

A summary of demographic data gathered for enrolled subjects and the participating caregivers can be found in Table 1. The average age of pediatric patients recruited in this study was 13.7 years, and the average age of caregivers was 46.2 years. Overall, 50% of the pediatric patients participating in the study were male and 50% were female. Surgical procedures performed as part of patients’ elective orthopedic surgery along with their diagnosis are listed in Table 2.

**Table 1:** Summary of participant demographics (n = 48).

Participant demographic	Subcategory	Mean	Range
Patient age		13.7	8–17
Parent age		46.2	37–74
		n	%
Patient gender	Male	24	50%
	Female	24	50%
Parent gender	Male	10	21%
	Female	38	79%
Parent relationship status	Married	43	91%
	Single (divorced/separated)	3	6%
	Single (widowed)	1	2%
Parent race	White	27	57%
	Asian	11	23%
	Hispanic	7	15%
	Multiracial	1	2%
	Other	1	2%
Insurance	Medicaid/Medi-Cal	11	23%
	Private	37	77%
Employment	Full-time	25	52%
	Part-time	5	10%
	No work outside home	11	23%
	Retired	5	10%
	Unemployed	2	4%
Education	Post-college graduate degree	14	29%
	4 year college degree	22	46%
	2 year college degree	3	6%
	High school	9	19%
Income	$50,000–$99,999	7	15%
	$100,000–$149,999	6	13%
	$150,000–$199,000	10	21%
	$200,000–$249,000	4	8%
	>$250,000	14	29%

**Table 2:** Elective orthopedic procedures with patient diagnosis.

Surgical procedure	Patient’s diagnosis
Achilles tendon lengthening	Achilles tendonitis, idiopathic toe-walking
Tarsal coalition excision	Tarsal coalition
Accessory navicular excision	Accessory navicular
Bunion repair	Foot bunion
Os trigonum excision	Achilles tendonitis
Tibialis anterior tendon transfer	Talipes equinovarus
Flatfoot reconstruction	Pes planus
Arthrocentesis	Subtalar joint fibrosis
Syndactyly reconstruction	Toe syndactyly
Proximal interphalangeal joint pinning	Hammertoe
Pelvic osteotomy	Acetabular dysplasia
Periacetabular osteotomy with iliac crest bone graft	Developmental dysplasia of the hip
Varus derotational intertrochanteric femoral osteotomy	Femoral anteversion
Distal femur epiphysiodesis	Leg length discrepancy
Open reduction and internal fixation of femoral shaft fracture	Monostotic fibrous dysplasia of the lower leg
Tibia/fibula osteotomy	Osteogenesis imperfecta type V
Knee arthroscopy with meniscectomy and/or meniscus repair	Meniscal tear of the knee, dislocation of the knee
Anterior cruciate ligament reconstruction	Anterior cruciate ligament rupture
Drilling of osteochondritis dissecans lesion and tibial tubercle ossicle resection	Osteochondritis dissecans of the knee
Open reduction and internal fixation of patella chondral fracture	Internal derangement of the knee
Ganglion cyst excision	Ganglion cyst
Orthopedic hardware removal	Retained orthopedic hardware, adolescent idiopathic scoliosis, proximal tibia fracture, perthes disease, inflammatory reaction to hardware
Bone cyst excision and curettage	Multiple exostoses syndrome, unicameral bone cyst
Anterior spinal fusion and instrumentation	Adolescent idiopathic scoliosis
Posterior spinal instrumentation and fusion	Juvenile idiopathic scoliosis, adolescent idiopathic scoliosis, neuromuscular scoliosis, scheuermann's kyphosis, spondylolisthesis
Transforaminal lumbar interbody fusion	Spondylolisthesis
Extreme lateral interbody fusion	Adolescent idiopathic scoliosis
Microdiscectomy	Lumbar disc herniation with radiculopathy

**Figure 1:**
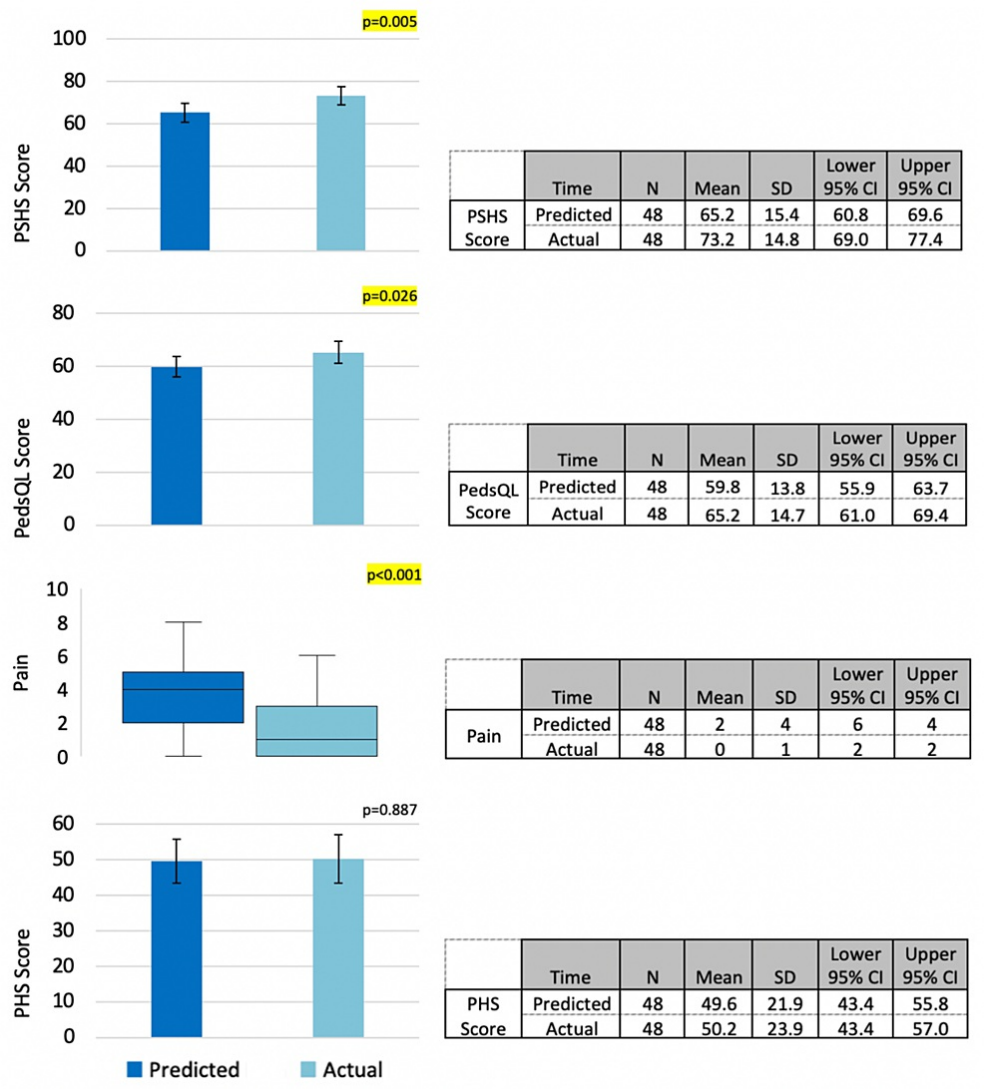
Comparison between caregiver’s prediction and child’s outcome. PSHS = Psychosocial Health Summary Score; PedsQL = Pediatric Quality of Life Inventory; PHS = Physical Health Summary Score

Caregivers underestimated their child’s postoperative psychosocial functioning, as evidenced by significantly decreased PSHS in comparison to their child’s actual scores, which were higher than predicted after undergoing surgery (p = 0.005) (Figure 1). Postoperative pain levels experienced by the child were also overestimated by caregivers, which was demonstrated by significant differences in comparative scores of the NRPS between patient and caregiver responses (p < 0.001) (Figure 1).

The PedsQL scores showed caregivers significantly underestimated their child’s overall quality of life (p = 0.026) after their procedure and had differing expectations of the impact surgery had across various aspects of the physical, emotional, social, and school functioning domains. Within the physical domain, caregivers underestimated the ability of the child to run (p < 0.001) and participate in sports/exercise activities (p < 0.001). They also overestimated the child experiencing any hurts/aches (p = 0.002) and being able to bathe independently (p < 0.001) after surgery. In the emotional domain, caregivers were found to overestimate their child experiencing feelings of being afraid/scared (p = 0.003), sad/blue (p = 0.042), and the extent to which they may worry about what will happen (p = 0.005). Among the social domain, caregivers overestimated their child’s potential to keep up when playing with other kids (p = 0.001) and getting along with other children (p = 0.002). For school functioning, caregivers overestimated their child missing school because of feeling unwell (p = 0.030), their ability to pay attention in class (p = 0.024), and their ability to keep up with schoolwork (p = 0.012). No significant difference was found in the patient’s postoperative physical functioning, as shown by the PHS (Figure 1).

**Figure 2:**
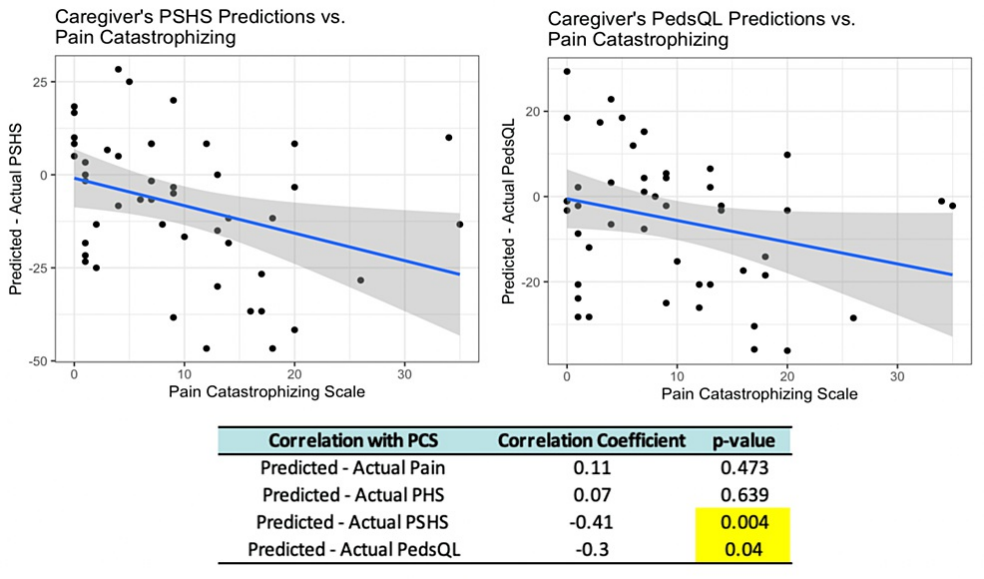
Correlation between parental pain catastrophizing and child’s postoperative functioning. PSHS = Psychosocial Health Summary Score; PedsQL = Pediatric Quality of Life Inventory; PHS = Physical Health Summary Score

Higher parental pain catastrophizing scores, as measured by the PCS, were associated with significantly underestimated predictions of their child’s psychosocial functioning (p = 0.004). Higher PCS scores were also found to be correlated to significant underestimations of the child’s overall quality of life (p = 0.040) after surgery (Figure 2). No significant difference was found between PCS scores and predictions of the child’s pain levels after surgery (p = 0.473). Additionally, there was no significant difference between PCS scores and caregiver’s expectations of their child’s physical functioning (p = 0.639).

## Discussion

The impact of psychosocial factors on one’s health has been an area of increasing study over the last several years, and even within musculoskeletal and orthopedic literature [5-9]. Studies among adult patients undergoing surgical orthopedic intervention have revealed that factors such as depression, anxiety, catastrophic thinking, and self-efficacy can affect a patient’s functional outcomes as variation in experiencing the intensity of symptoms has been attributed to one’s mental and social health [3,5,6,13]. Similar correlations are yet to be investigated among the pediatric population where the interaction between parents and their children, and behaviors such as pain catastrophizing, caregiver protective response, and familiar environment, can affect a child’s experienced symptoms and outcomes of treatment [10-12]. This further highlights the need to understand the differences between caregiver predictions and the child’s actual postoperative outcomes to create opportunities for targeted education.

Encouraging open discussion between healthcare providers and caregivers about the child’s expected postoperative outcomes begins with identifying typical areas that caregivers inaccurately estimate the effect surgery will have on the child and their functioning. In our study, caregivers underestimated their child’s postoperative psychosocial functioning and overall quality of life after surgery and overestimated their child’s post-procedure pain levels. Parents also had varied perceptions about the impact of surgery on certain aspects of the child’s performance across the physical, emotional, social, and school functioning domains. Given the extent to which caregivers believe orthopedic surgery may affect various facets of their child’s life, prioritizing early discussions about realistic expectations of pain and function becomes imperative to mitigate parental concerns and anxiety. Areas of discrepancy in predictions of the child’s abilities after orthopedic surgery provide valuable insights into particular realms to be proactively addressed in supporting families through a child’s surgical orthopedic care.

In addition to healthcare provider counseling and support when addressing specific domains of the child’s postoperative functioning, parents with higher scores of pain catastrophizing, or a heightened negative perception of anticipated events, may benefit from focused intervention [11]. Research on maladaptive patterns of cognition demonstrates a correlation between catastrophic thinking and the intensity of symptoms when inaccurate expectations of an experience are interpreted as reality [3,14]. In our study, we found that caregivers with higher pain catastrophizing underestimated the child’s overall psychosocial functioning and quality of life after surgery. Other studies investigating the association between parental catastrophizing and pediatric outcomes have demonstrated correlations between increased levels of caregiver catastrophizing with adverse psychosocial outcomes, increased depression and anxiety, and higher functional disability among children with chronic pain [15,16]. Catastrophizing has also been implicated in evoking protective responses from the caregiver in an attempt to relieve the child’s symptoms, which can prolong the duration of pain, depression, and functional disability in the child [17-19]. Therefore, parents who are found to demonstrate higher catastrophizing may be candidates for targeted programs designed to promote positive and adaptive coping mechanisms.

This study has limitations that are important to note, including a small sample size and convenience sampling which may affect the generalizability of results. Additionally, as patients were undergoing elective orthopedic surgery with a planned procedure, findings may vary for children who require urgent or emergent care. The length and complexity of the procedure that children endure may also affect various factors in this study. Further research as an extension of our study efforts may investigate effective modalities to provide targeted information and education, and whether the age of the child at the time of procedure or the type of procedure endured affects the extent to which parental catastrophizing may impact their outcomes. Additionally, evaluating whether proactive counseling about the effect surgery may have on a child in the early postoperative period affects parental catastrophizing and patient outcomes can provide insights into whether prioritizing these discussions before surgical treatment is worthwhile.

## Conclusions

It is evident that a complex interplay exists between emotional and behavioral parental responses that both directly and indirectly impact their child. Explaining to caregivers that the child’s psychosocial and functional outcomes after orthopedic surgery are commonly better than expected may help prevent catastrophizing and alleviate anxiety. This knowledge can also help guide caregiver expectations and plans for their child’s postoperative pain control and functional recovery. Taking additional time to counsel parents and caregivers in the preoperative period about expectations of their child’s postoperative functioning may be beneficial in alleviating anxiety and concerns that can contribute to increased catastrophizing. The implementation of such interventions can provide an added level of support for both the parent and the child as they progress through surgical treatment and recovery.
